# Use of Healthcare Services Two Years before Diagnosis in Danish Sarcoma Patients, 2000–2013

**DOI:** 10.1155/2019/8108590

**Published:** 2019-05-07

**Authors:** Mathias Rædkjær, Katja Maretty-Kongstad, Thomas Baad-Hansen, Akmal Safwat, Michael M. Petersen, Johnny Keller, Peter Vedsted

**Affiliations:** ^1^Department of Orthopaedic Surgery, Aarhus University Hospital, Palle Juul-Jensens Boulevard 99, 8200 Aarhus N, Denmark; ^2^Department of Orthopaedic Surgery, Rigshospitalet, Blegdamsvej 9, 2100 Copenhagen, Denmark; ^3^Department of Experimental Clinical Oncology, Aarhus University Hospital, Palle Juul-Jensens Boulevard 99, 8200 Aarhus N, Denmark; ^4^Department of Pathology, Aarhus University Hospital, Palle Juul-Jensens Boulevard 99, 8200 Aarhus N, Denmark; ^5^Department of Oncology, Aarhus University Hospital, Palle Juul-Jensens Boulevard 99, 8200 Aarhus N, Denmark; ^6^The Research Unit of General Practice, Aarhus University, Bartholins Allé 2, 8000 Aarhus C, Denmark; ^7^Silkeborg Hospital, Department of Clinical Medicine, Aarhus University, Falkevej 1G, 8600 Silkeborg, Denmark

## Abstract

**Background:**

Sarcoma is a rare type of cancer with nonspecific symptoms and uncertain aetiology. Thus, timely diagnosis of sarcomas is a clinical challenge. The aim of this study was to investigate the use of healthcare services 24 months preceding a sarcoma diagnosis compared to a matched cohort.

**Materials and Methods:**

The study was a retrospective, population-based, matched cohort registry-study. Patients with sarcoma in Denmark in 2000–2013 were identified in the Danish Sarcoma Registry (*n* = 2167) and matched 1 : 10 on gender, age, and listed general practice. Using a binomial regression model, incidence rate ratios were calculated for face-to-face contacts in general practice, inpatient and outpatient visits, surgery, paraclinical examinations, and diagnostic imaging. Analyses were stratified for sarcoma subtypes, grade, stage, gender, and presence of comorbidity.

**Results:**

The sarcoma patients had significantly increased incidence rate ratios in use of healthcare services compared to the matched cohort a year before their diagnoses. An increase in consultation rates was seen 11 months before diagnosis for inpatient visits, 9 months before diagnosis in general practice and outpatient visits, 8 months before diagnosis for paraclinical examinations, and 4 and 3 months before diagnosis for diagnostic imaging and surgery, respectively. There were no clinical significant differences in length of increased consultation rates between sarcoma type, stage, and grade. Sarcoma patients with comorbidity had persistently higher consultation rates compared to patients without comorbidity.

**Conclusions:**

The use of healthcare services among sarcoma patients increased several months before diagnosis in all healthcare sectors. The results reveal a diagnostic time window and a potential to refer, diagnose, and treat sarcoma patients in a timelier manner.

## 1. Introduction

Sarcoma is a rare cancer representing 1% of all newly diagnosed malignancies in Denmark [1]. The aetiology is generally unknown, and the symptoms mimic many benign conditions. Delay of diagnosis is well known in sarcomas and is a recognised problem for many other types of cancer [2–9]. An earlier diagnosis may have an impact on the size and stage at diagnosis and thus improve outcome, though different studies point towards contradictory results [3, 10–12]. The introduction of fast-track cancer referral pathways seems to have reduced the time from referral and until the sarcoma is finally diagnosed [13]. The largest part of waiting time before referral to a sarcoma centre can be ascribed to the patient and the local hospital [14]. Thus, the key to reduce time from symptom to treatment might lie in the time before the patient gets referred to a sarcoma centre [13].

In Denmark, 98% of the residents are listed with a general practice [15]. The GPs act as gatekeepers to the rest of the healthcare system and play an important role in preventive healthcare, screening, diagnosis, treatment, and referral to practicing specialists or hospitals. The Danish healthcare system is tax-funded, and ensures a uniform, free access to healthcare services regardless of socioeconomic position [15]. The aspects of low incidence, nonspecific symptom presentation, and low symptom awareness may delay the diagnostic process. Outlining diagnostic activity in use of healthcare services before sarcoma diagnosis is therefore a crucial first step in any effort aimed at an early detection of the disease [2, 3, 5, 16, 17].

The aim of this study was to investigate the use of different healthcare services 24 months preceding a sarcoma diagnosis compared to a matched cohort, stratified for sarcoma subtype, grade, stage, gender, and presence of comorbidity.

## 2. Materials and Methods

### 2.1. Study Design and Study Population

This nationwide population-based matched comparative study was performed using data from Danish national registries. Treatment of sarcomas in Denmark is centralised in two centres in Aarhus and Copenhagen. All sarcoma patients are diagnosed and treated according to national guidelines by an experienced multidisciplinary sarcoma team [18, 19].

From the Danish Sarcoma Registry (DSR), we identified patients ≥15 years of age with a histopathologically verified soft tissue or bone sarcoma located in the extremities or trunk wall diagnosed from 1 January 2000 till 31 December 2013 (*n* = 2167). The index date was defined as the date of the final pathology report and the histopathological diagnosis of the sarcoma. The date of diagnosis was chosen based on the hierarchy produced by the European Network of Cancer Registries [20]. The DSR is a validated, national, population-based clinical database containing all patients with sarcoma in the extremities or trunk wall from 2000 onwards and holds information on patient characteristics and detailed data on tumour characteristics, surgical and oncological treatment, local and distant recurrences, and death [21, 22].

All Danish residents are assigned a unique civil registration number (CPR-number) at birth or immigration and appear in the Civil Registration System (CRS). This enabled us to link all data from the DSR and the different Danish national registries on an individual level [23].

For each sarcoma patient, 10 references matched on age (±12 months), gender, and listed general practice were identified through CRS and the Patient List Registry (PLR). The PLR is an administrative registry where information on any person registered with a general practice at a given time is available [15]. Index date was defined as the day of diagnosis of the sarcoma patient. Cases or references living outside of Denmark at some point during the 24-month period before the index date were ineligible.

A range of healthcare services were selected to estimate the prediagnostic healthcare activity for both cases and references. Data from consultations in general practice, public and private hospitals, and practicing specialists were retrieved from the National Health Insurance Registry (NHSR) and the National Patient Registry (NPR). All Danish healthcare services are registered prospectively in NHSR with the purpose of remuneration, and data are therefore practically complete [24]. Admissions, discharge dates, ICD-10 diagnoses, and data on imaging were obtained from the NPR. The NPR is a national population-based database with a mandatory obligation to report and covers more than 99% of all Danish hospital admissions [25].

For general practice, monthly rates of face-to-face contacts in daytime were calculated, including normal consultations and home visits and excluding specific preventive consultations and out-of-hours. It was not possible to gain information regarding reason and the content of the consultations. For inpatient visits (patients hospitalized for an undefined period of time and registered as such), outpatient visits (patients referred to a hospital on an ambulant/outpatient basis, e.g., directly by the GP), and surgery (registered surgical procedures), we obtained all discharge diagnoses within the specialities orthopaedic surgery, dermatology, and plastic surgery. Paraclinical examinations ordered by the same range of specialities were obtained as well. Diagnostic imaging was obtained by extracting data on registered conducted services from all public and private departments of radiology.

All of the consultation rates were independent of whether the patient had symptoms of sarcoma or not 24 months prior to their sarcoma diagnosis.

Based on the Charlson Comorbidity Index (CCI) score obtained from the NPR 10 years prior to the sarcoma diagnosis, we divided comorbidity into “no comorbidity” with a CCI score of 0 and “comorbidity” with a CCI score of ≥1 [26].

Data on tumour characteristics (subtype, grade, stage, and tumour size) were obtained from the DSR. The patients were divided into groups depending on the subtype of sarcoma (soft tissue sarcoma and bone sarcoma), according to stage (localised or disseminated disease), grade, and size, at time of diagnosis. Low-grade sarcoma was defined as grade I and high-grade sarcoma as grade II + III, according to the histopathological grading system [27]. The cutoff values regarding size were defined as large if soft tissue sarcoma exceeded 5 cm and bone sarcoma 8 cm, according to the WHO Classification of Tumours of Soft Tissue and Bone, fourth edition, and the TNM classifications [28, 29].

### 2.2. Statistical Analysis

In order to compare the monthly contact rates, the incidence rate ratios (IRRs) were calculated using a binomial regression model applying robust variance due to clusters of patients [30]. All IRRs presented are adjusted for age and gender. All tests were two-sided, and *p* values of 0.05 or less were considered statistically significant. Estimates were made with corresponding 95% confidence intervals (CI). All analyses were performed in STATA 14.2 software.

### 2.3. Ethical Approval

The Danish Data Protection Agency (j.nr: 1-16-02-245-14), Statens Serum Institute (FSEID-1729), Statistics Denmark (p.nr: 706699), and the Danish Clinical Registries (j.nr: DSD-2017-03-02) approved this study.

## 3. Results

### 3.1. Characteristics of Study Population and Reference Population

A total of 2167 sarcoma patients (cases) and 21,670 references were included in this study. Characteristics of the sarcoma cohort and reference population are listed in Table 1.

### 3.2. Use of Healthcare Services before Diagnosis

**Figure 1:**
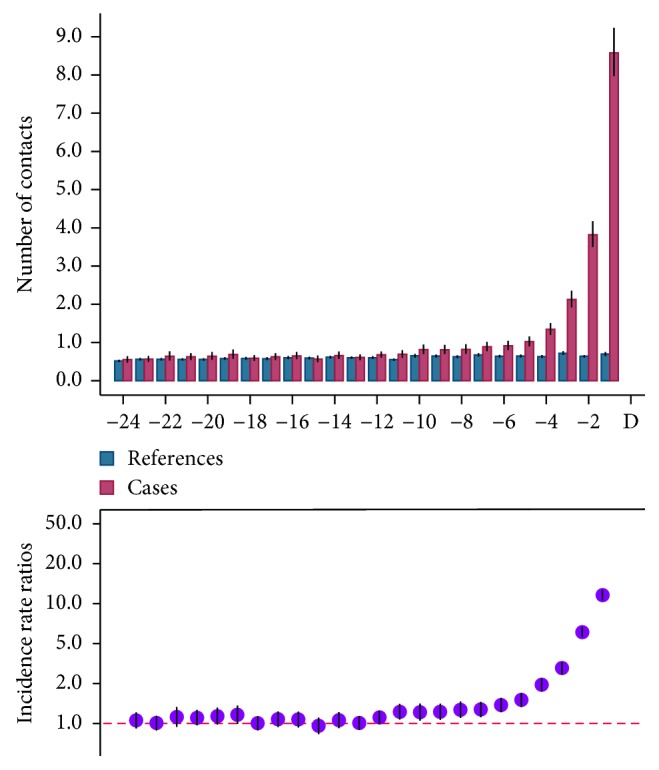
Monthly rates and incidence rate ratios (IRRs) for the total use of healthcare services for references and cases 24 months before the sarcoma diagnosis, with 95% confidence interval (CI).

There was a clear increase in overall consultation and examination rates in the period before the sarcoma diagnosis in the study population compared to the reference group (Figure 1). The IRRs were statistically significant in the 12 consecutive months preceding diagnosis with a peak in the final month with an IRR of 13.89 (CI 12.41–15.54).

**Figure 2:**
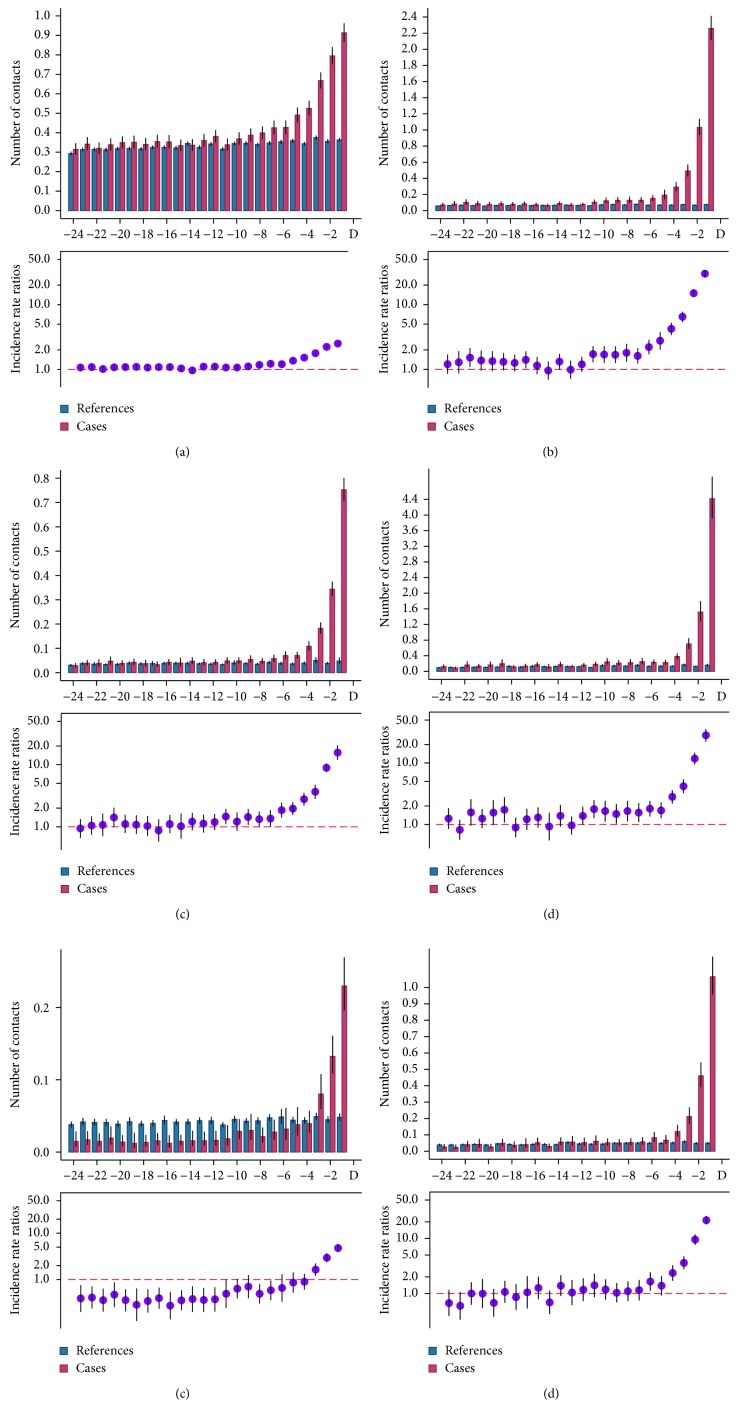
Monthly rates and incidence rate ratios (IRRs) for use of health care services among references and cases in (a) general practice, (b) inpatient visits, (c) outpatient visits, (d) paraclinical examinations, (e) surgery, and (f) diagnostic imaging for cases and references 24 months before the sarcoma diagnosis, with 95% confidence interval (CI). Note the differences in the *Y*-axis range.

Face-to-face consultation rates in general practice are shown in Figure 2(a). The IRRs were consecutively statistically significantly higher from 9 months prior to diagnosis with a peak in the last month. The rates of contacts in general practice increased significantly from 9 months prior to diagnosis, and general practice was far most the place where the patients had most contacts. Inpatient and outpatient visits are shown in Figures 2(b) and 2(c).

Statistically significant increases in IRRs were seen from 11 to 9 months prior to diagnosis, respectively.

The most pronounced difference between the case population and the references in the month before diagnosis was in paraclinical examinations with an IRR of 33.1 (Figure 2(d)). Diagnostic imaging had statistically significant IRRs 4 months prior to diagnosis (Figure 2(f)).

### 3.3. Use of Healthcare Services in Different Subgroups of Sarcoma Patients

**Figure 3:**
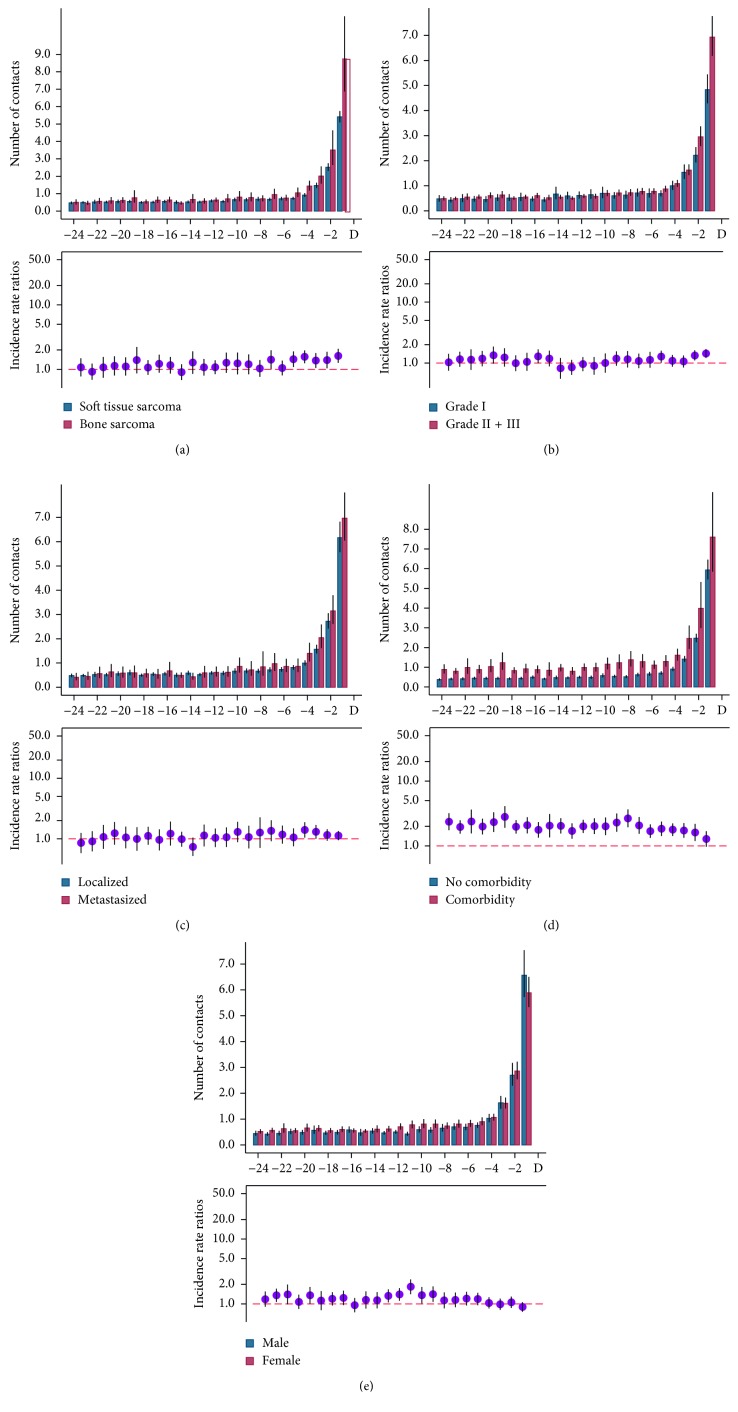
Monthly rates and incidence rate ratios (IRRs) for use of healthcare services in the case population in (a) sarcoma type, (b) tumour grade, (c) tumour stage, (d) comorbidity, and (e) gender for cases and references 24 months before the sarcoma diagnosis, with 95% confidence interval (CI). Note the differences in the *Y*-axis range.

The overall rates of healthcare services were higher among bone sarcoma patients compared to soft tissue sarcoma patients, and the IRRs were statistically significantly increased in the last four months before diagnosis (Figure 3(a)).

The overall use of healthcare services was very similar between high- and low-grade sarcoma until the last 3 months, where high-grade sarcoma had statistically significantly higher IRR (Figure 3(b)). There was no statistically significant difference in IRRs for patients with localised disease compared to patients with disseminated disease at the time of diagnosis (Figure 3(c)).

For all 24 months, the IRRs in sarcoma patients with comorbidity were statistically significantly increased except for the last month before diagnosis (Figure 3(d)).

Women had statistically significantly higher IRR rates in 5 scattered months before diagnosis though no statistically significant IRRs were found in the last 8 months before diagnosis (Figure 3(e)).

## 4. Discussion

### 4.1. Main Findings

This nationwide, population-based study of 2167 sarcoma patients showed a marked increase in use of healthcare services in the period leading up to the sarcoma diagnosis when compared to a 1 : 10 matched cohort. In general, an increasing number of contacts were observed from about one year before a sarcoma diagnosis.

The rates of contacts in general practice increased significantly from 9 months prior to diagnosis. This may be due to the gatekeeper role and using “wait-and-see” before referring to further investigation. The use of healthcare services in hospital started at nearly the same time about 8 months before which may be due to GPs referring the patient and an increased use of casualty departments. There was a tendency that bone sarcomas were seen more often in the months leading up to diagnosis compared to soft-tissue sarcomas, which reflects the symptomatology, which may mimic more benign conditions (musculoskeletal complaints).

### 4.2. Strengths and Limitations

Information on all the contacts in the healthcare system was collected through nationwide Danish registries with a high level of accuracy and completeness [31]. This is due to the mandatory obligation to report events and procedures at the hospitals and that the registration is the basis for remuneration of the GPs and private practicing specialists. Sarcomas are rare, but by using data from DSR, we obtained complete population-based, nationwide data [21, 22]. Selection bias and information bias in relation to healthcare services would be negligible, since the data were not collected for the purpose of this study. To minimise the risk of confounding, the references were matched according to age, gender, and general practice. By matching on general practice, the GP-dependent and area-specific differences in healthcare seeking were reduced. The hospital departments and private practicing specialists of interest were chosen from a clinical point of view. The hypothesis was that a GP might refer to an orthopaedic surgeon, a dermatologist, a plastic surgeon, or a physiotherapist taking the characteristic symptoms of sarcomas into consideration.

A limitation of this study in terms of qualifying the number of consultations is the lack of knowledge regarding the reason and the content of the consultations in both cases and references and the indication for the referrals. Furthermore, the 24 months prior to diagnosis included the use of healthcare services, i.e., diagnostic imaging, paraclinical examinations, and biopsies ordered by the department at the sarcoma referral centre, and hence, the cutoff in diagnostic workup before and after suspected sarcoma may be vague.

### 4.3. Comparison with Other Studies

The statistically significant increase in consultation rates in general practice about 9 month before diagnosis may be due to the nonspecific symptom presentation in sarcoma patients and low incidence, which makes awareness among clinicians and patients lower. In other types of cancer, the consultation rate in general practice prior to diagnosis varied quite extensively [8, 32–35]. Diseases with low incidence and uncharacteristic symptoms, such as CNS tumours, have a longer period of increased consultation rates in general practice with 17 months before diagnosis, than malignant melanoma with only three months before diagnosis [33]. It must be noted that the patients already, and in particular those with comorbidity, accessed general practice as the most used healthcare service.

Parallel with the increased use of general practice, there was a significant increase in use of inpatient and outpatient visits and paraclinical tests. This could likewise be due to the vague symptoms and low awareness of the disease [14]. Further, it could be an expression of ongoing diagnosis of the sarcoma, since the general practitioner—in case of a suspected sarcoma—would refer to the local hospital in the first place. Still, diagnostic imaging and paraclinical examinations had the latest onset of activity, about four months before diagnosis, which could indicate that the sarcoma diagnosis is suspected at this point.

There was a tendency of increased consultation rates among patients with bone sarcomas compared to those with soft tissue sarcomas. One of the main symptoms of bone tumours is an increasing pain over weeks, which could be part of the reason [36]. As such, being wary of persistent deep pain from a bone, especially in children and young adults, is important.

Sarcoma patients with comorbidity had higher consultation rates compared to the sarcoma patients without comorbidity, which may reflect the habitual difference in rates of consultations in patients with multimorbidity in the general population [37, 38]. During the months prior to diagnosis, the difference (IRR) between patients with or without comorbidity decreased, indicating that the diagnostic workup has the same contents in the two groups. Comorbidity may mask early cancer symptoms and thus increase the diagnostic delay and lead to a more advanced stage by the time the patient is referred to a sarcoma centre [39]. On the contrary, higher prevalence of comorbidity in early-stage cancers has been described in other cancers [40, 41]. GPs and other health professionals should have in mind that patients with cancer and comorbidity have a poorer prognosis and thus be aware of multimorbid patients with aberrant symptoms in order of timely referral [25, 42, 43].

The period of increased contacts among sarcoma patients before diagnosis in general practice is long compared to other types of cancer [33, 44]. Other studies have found a median diagnostic interval of 14–16 months in sarcoma patients [2, 45, 46]. In a study of paediatric and adolescent soft tissue sarcoma patients, the symptom interval ranged between 1 week and 60 months and had a negative impact on survival [47]. The introduction of cancer fast-track referral pathways in Denmark has facilitated a more expedited diagnosis, and the known defined alarm symptoms were predictive for sarcoma patients but had a low positive predictive value in general [13].

One way to reduce the diagnostic interval could be to give the GP the opportunity to refer directly to a MRI or CT scan for suspected sarcomas, instead of referring to a local hospital as today. This has proven to reduce specialist time in lung cancer patients [48]. The advantage is a shorter route to a sarcoma centre if the scan confirms the suspicion of the sarcoma diagnosis, but without the concern among the patients that go through a fast-track cancer patient pathway. The disadvantage is the risk of overburdening the radiology departments, as indicated in previous studies of other cancer types [48–50]. The study of lung cancer patients demonstrated that direct referral to CT scan did not cause an increase in the number of CTs performed [48].

Together with better access to imaging, a short relevant update on sarcomas and early diagnosis, timely referral, and how to select patients for further examination might be relevant. This has proven to be relevant in lung cancer [51].

## 5. Conclusion

This study revealed an increased use of healthcare services and diagnostic activity in both primary and secondary care for Danish sarcoma patients in the years leading up to the diagnosis. Increased contacts in general practice started nine months before diagnosis and coincided with an increased use of imaging and hospitals. This reflects a diagnostic window where general practice is dependent on a responsive health care system. The results indicate that a diagnostic time window is present, and the potential to refer, diagnose, and treat sarcoma patients in a timelier manner exists.

Further studies could focus on the content of the consultations and the indications for and access to further examinations.

## Figures and Tables

**Table 1:** Patient characteristics for 2167 sarcoma patients diagnosed from 2000 to 2013 in Denmark and 21,670 references matched on age, gender, and general practice.

	Cases	References
	*N* (%)	*N* (%)
Total	2167 (100)	21670 (100)
Age (years)		
Median (range)	56 (15–96)	56 (14–101)
15–49	759 (35)	7576 (35)
50–69	823 (38)	8297 (38)
≥70	584 (27)	5797 (27)
Gender		
Female	972 (45)	9752 (45)
Male	1195 (55)	11918 (55)
Sarcoma type		
Soft tissue sarcoma	1617 (76)	—
Bone sarcoma	509 (24)	—
Stage at diagnosis		
Localised	1906 (88)	—
Disseminated	261 (12)	—
Grade at diagnosis		
Low grade (I)	509 (26)	—
High grade (II + III)	1482 (74)	—
Comorbidity		
No comorbidity	1741 (80)	—
Comorbidity	426 (20)	—

*N*: number. Sarcoma type: 41 missing. Grade: 176 missing.

## Data Availability

Data from the Danish national registries (the Danish Sarcoma Registry, the National Patient Registry, the Civil Registration System, and the Cause of Death Registry) are only available for researchers and institutions who meet the criteria for access to confidential data. All data belong to third party and are not owned by the authors. Future researchers will be able to access the data through the same process by which the authors of this manuscript did. Authorisation to manage and process data is given by Danish Data Protection Agency (https://www.datatilsynet.dk/english/; e-mail: dt@datatilsynet.dk). Data are applicable from the Danish Sarcoma Registry (http://www.rkkp.dk/in-english/; http://www.rkkp.dk/forskning/; e-mail: fagligkvalitet@rkkp.dk), the National Patient Registry, the Civil Registration System, and the Cause of Death Registry (https://sundhedsdatastyrelsen.dk/da/forskerservice/ansog-om-data; e-mail: kontakt@sundhedsdata.dk).
